# Physiological and morphological differences of airways between COPD and asthma–COPD overlap

**DOI:** 10.1038/s41598-019-44345-6

**Published:** 2019-05-24

**Authors:** Masato Karayama, Naoki Inui, Hideki Yasui, Masato Kono, Hironao Hozumi, Yuzo Suzuki, Kazuki Furuhashi, Dai Hashimoto, Noriyuki Enomoto, Tomoyuki Fujisawa, Yutaro Nakamura, Hiroshi Watanabe, Takafumi Suda

**Affiliations:** 1 Second Division, Department of Internal Medicine, Hamamatsu University School of Medicine, 1-20-1 Handayama, Hamamatsu, 431-3192 Japan; 2 Department of Clinical Pharmacology and Therapeutics, Hamamatsu University School of Medicine, 1-20-1 Handayama, Hamamatsu, 431-3192 Japan

**Keywords:** Chronic obstructive pulmonary disease, Three-dimensional imaging, Asthma

## Abstract

Overlap of asthma and COPD has attracted attention recently. We aimed to clarify physiological and morphological differences of the airways between COPD and asthma–COPD overlap (ACO). Respiratory resistance and reactance and three-dimensional computed tomography data were evaluated in 167 patients with COPD. Among them, 43 patients who fulfilled the diagnosis of asthma were defined as having ACO. Among 124 patients with COPD without ACO, 86 with a comparable smoking history and airflow limitation as those with ACO were selected using propensity score matching (matched COPD). The intraluminal area (Ai) and wall thickness (WT) of third- to sixth-generation bronchi were measured and adjusted by body surface area (BSA; Ai/BSA and WT/√BSA, respectively). Patients with ACO had higher respiratory resistance and reactance during tidal breathing, but a smaller gap between the inspiratory and expiratory phases, compared with matched patients with COPD. Patients with ACO had a greater WT/√BSA in third- to fourth-generation bronchi, smaller Ai/BSA in fifth- to sixth-generation bronchi, and less emphysematous changes than did matched patients with COPD. Even when patients with ACO and those with COPD have a comparable smoking history and fixed airflow limitation, they have different physiological and morphological features of the airways.

## Introduction

Chronic obstructive pulmonary disease (COPD) has fixed airflow limitation (mostly irreversible, but partially reversible in some cases), which is observed in current or former smokers of middle and old age^1^. Asthma, another obstructive lung disease, is characterized by recurring respiratory symptoms and variable expiratory flow limitation, which is partially or fully reversible with bronchodilators^2^. Differential diagnosis between COPD and asthma is easy when they have typical features of each disease. However, in clinical practice, many patients have clinical features of both asthma and COPD, which makes distinguishing obstructive lung diseases difficult. Recently, having features of both asthma and COPD has attracted attention as asthma–COPD overlap (ACO)^3,4^. Recognition of ACO is especially important for initial treatment because of some contraindications between ACO and COPD. Inhaled corticosteroids (ICSs) in combination with long-acting beta-agonists (LABAs) and/or long-acting muscarinic antagonists (LAMAs) are recommended in ACO, whereas LABA monotherapy is not. In contrast, pharmacotherapy with inhaled bronchodilators, LABAs, and LAMAs, alone or in combination, is the mainstay of COPD. ICSs are recommended for patients at high risk of exacerbations and with a history of frequent exacerbation events, and ICS monotherapy is not recommended^4^.

Despite the clinical significance, there is no universal definition of ACO. This is because patients with overlapping features were excluded from clinical studies for either asthma or COPD, and the characteristics of ACO have been poorly studied. Assessment of overlapping features using epidemiological, phenotypic, morphological, and physiological analysis is important for understanding the difference and/or overlap of these two diseases.

The diagnosis and severity of asthma and COPD are based on airflow limitation as assessed by spirometry, which is the most widely used physiological function test^1,2^. However, spirometry can provide limited information regarding discrimination of the overlap of these two diseases. Recently, assessment of respiratory impedance has attracted attention as a novel physiological test in several respiratory diseases, including asthma and COPD^5–12^. Respiratory impedance, which comprises resistance and reactance of the respiratory system, provides additional information of respiratory function of small airways^13–16^. Respiratory impedance can detect subtle changes in the airways in asthma and COPD, which are potentially more sensitive than those with conventional spirometry^5,6,10–12^. Even healthy smokers with normal spirometry have impaired respiratory impedance, indicating early remodelling of the small airways^17^. Measurements of respiratory impedance could be a useful approach for clarifying the characteristics of ACO.

Computed tomography (CT) of the chest provides useful information on structural changes in the airways. Airway changes in asthma and COPD that are assessed by CT analyses correlate with pathological abnormality, physiological impairment, and response to inhaled therapy^18–22^. Airway changes as assessed by CT are associated with airflow limitation in non-COPD smokers^20^. Additionally, three-dimensional (3D) CT analysis provides more detailed information on the airways in asthma and COPD^23,24^. Analysis of 3D-CT of the airways may provide novel information for understanding ACO.

Using these modalities, we aimed to clarify the differences in physiological and morphological airway changes between patients with COPD and those with ACO. We evaluated respiratory impedance, which was measured by the forced oscillation technique, and airway structure, which was measured by 3D-CT analyses. We adjusted for patients’ demographic characteristics using propensity score-matched analyses.

## Results

### Patients’ characteristics

Forty-three patients were identified as having ACO. These patients had significantly lower pack-year smoking (*p* = 0.005) and a higher body mass index (BMI, *p* = 0.036) compared with 124 patients with COPD (Supplementary Table S1). To minimize bias between patients with ACO and COPD, matched patients with COPD were selected by propensity score-matched analysis in one-to-two matching using age, sex, BMI, and pack-year smoking. After propensity score matching, 86 matched patients with COPD were selected who had comparable characteristics with those with ACO (Table 1). The percentages of the Global Initiative for Chronic Obstructive Lung disease (GOLD) severity of airflow limitation I, II, III, and IV were not significantly different between the ACO and matched COPD groups (*p* = 0.784). Among patients with ACO, seven (16.3%), three (7.0%), four (9.3%), 23 (53.5%), and two (4.7%) received GINA-recommended treatment steps of 1, 2, 3, 4 and 5, respectively. Thirty-six (83.7%) patients with ACO received an ICS (fluticasone equivalent median [range] dose was 320 [100–1000] μg). Patients with ACO more frequently received ICSs (*p* < 0.001) and LABAs (*p* = 0.060), and received less LAMAs (*p* < 0.001) compared with matched patients with COPD.

**Table 1:** Patients’ characteristics.

	ACO (n = 43)	Matched COPD (n = 86)
Age, years	69.3 (7.7)	70.1 (9.3)
Sex: male	36 (83.7)	80 (93.0)
**Smoking status**		
Current smoker	5 (11.6)	18 (20.9)
Former smoker	38 (88.4)	68 (79.1)
Pack-year	39.8 (37.2)	45.7 (32.1)
BMI (kg/m^2^)	24.2 (4.6)	23.2 (2.9)
**Pulmonary function tests**		
FVC, % predicted	91.8 (13.7)	96.6 (16.7)
FEV_1_, % predicted	69.4 (19.0)	70.3 (20.3)
FEV_1_/FVC (%)	60.6 (13.9)	58.2 (12.9)
FEF_25–75_, % predicted	34.3 (18.8)	33.2 (17.8)
**GOLD stage**		
I	12 (27.9)	27 (31.4)
II	24 (55.8)	46 (53.5)
III	4 (9.3)	10 (11.6)
IV	3 (7.0)	3 (3.5)
**Treatment**		
ICS	36 (83.7)	2 (2.3)*
LABA	31 (72.1)	47 (54.7)
LAMA	11 (25.6)	54 (62.8)*

Data are expressed as number (%) or mean (standard deviation). ACO, asthma–COPD overlap; COPD, chronic obstructive pulmonary disease; BMI, body mass index; FVC, forced vital capacity; FEV_1_, forced expiratory volume in 1 second; FEF, forced expiratory flow rate; GOLD, Global Initiative for Chronic Obstructive Lung Disease; ICS, inhaled corticosteroid; LABA, long-acting beta-agonist; LAMA, long-acting muscarinic antagonist. ^§^Data are expressed as fluticasone equivalent among patients who received ICS. *p < 0.001 compared with ACO.

### Analysis of 3D-CT

**Figure 1:**
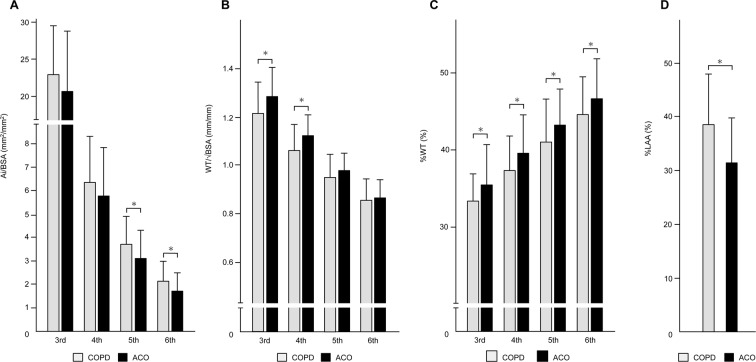
Comparison of three-dimensional computed tomography analyses of the airways between COPD and ACO. (**A**) Airway inner luminal area adjusted by body surface area (Ai/BSA). (**B**) Airway wall thickness adjusted by body surface area (WT/√BSA). (**C**) Percentage of airway wall thickness (%WT). (**D**) Percentage of low attenuation area <−950 HU (%LAA). COPD, chronic obstructive pulmonary disease; ACO, asthma–COPD overlap. The data are shown from third- to sixth-generation bronchi in sequential order. Grey and black bars indicate COPD and ACO, respectively.

Patients with ACO had a significantly lower airway inner luminal area adjusted by body surface area (Ai/BSA, mm^2^) from fifth- to sixth-generation bronchi (*p* = 0.018 and *p* = 0.004, respectively), higher wall thickness adjusted by square root of body surface area (WT/√BSA, mm) from third- to fourth-generation bronchi (*p* = 0.003 and *p* = 0.001, respectively), higher percentage of WT (%WT) from third- to sixth-generation bronchi (*p* = 0.023, *p* = 0.015, *p* = 0.017, and *p* = 0.033, respectively), and lower percentage of low attenuation area (%LAA, *p* < 0.001), compared with matched patients with COPD (Fig. 1, Supplementary Table S2). Variable clustering analysis selected Ai/BSA in fifth-generation bronchi, WT/√BSA in third-generation bronchi, and %LAA as representative variables of 3D-CT parameters (Supplementary Table S3). In multivariate logistic regression analysis, a higher WT/√BSA in third-generation bronchi and lower %LAA were significant predictive factors for ACO (Table 2). A cut-off value for the diagnosis of ACO was 1.23 mm in WT/√BSA in third-generation bronchi (sensitivity, 0.74; specificity, 0.63; and area under the receiver operating characteristic curve (AUC), 0.67) and 32.1% in %LAA (sensitivity, 0.67; specificity, 0.72; and AUC, 0.70). When using the second most representative variables, Ai/BSA in sixth-generation bronchi (instead of fifth-generation), WT/√BSA in fourth-generation bronchi (instead of third-generation), and %LAA were significant predictive factors for ACO (Supplementary Table S4). The results of univariate regression analyses for other 3D-CT variables are shown in Supplementary Table S5.

**Table 2:** Logistic regression analyses of 3D-CT for ACO.

Variables	Univariate		Multivariate	
	Odds ratio	*p*-value	Odds ratio	*p*-value
Age, per 1-year increase	0.99 (0.95–1.03)	0.638	0.99 (0.94–1.04)	0.662
Sex: female	2.59 (0.81–8.58)	0.107	2.47 (0.63–10.22)	0.194
3rd-WT/√BSA, per 0.1 mm/mm increase	1.56 (1.16–2.15)	0.005	1.59 (1.12–2.32)	0.009
5th-Ai/BSA, per 1 mm^2^/mm^2^ increase	0.68 (0.48–0.93)	0.020	0.78 (0.53–1.12)	0.181
%LAA, per 1% increase	0.92 (0.88–0.96)	<0.001	0.92 (0.88–0.96)	<0.001

Data are expressed as odds ratios (95% confident intervals). Ai, airway inner luminal area; WT, airway wall thickness; %LAA, percentage of low attenuation area <−950 HU; BSA, body surface area; 3rd-, third generation bronchi, 5th-, fifth generation bronchi.

### Respiratory impedance

**Figure 2:**
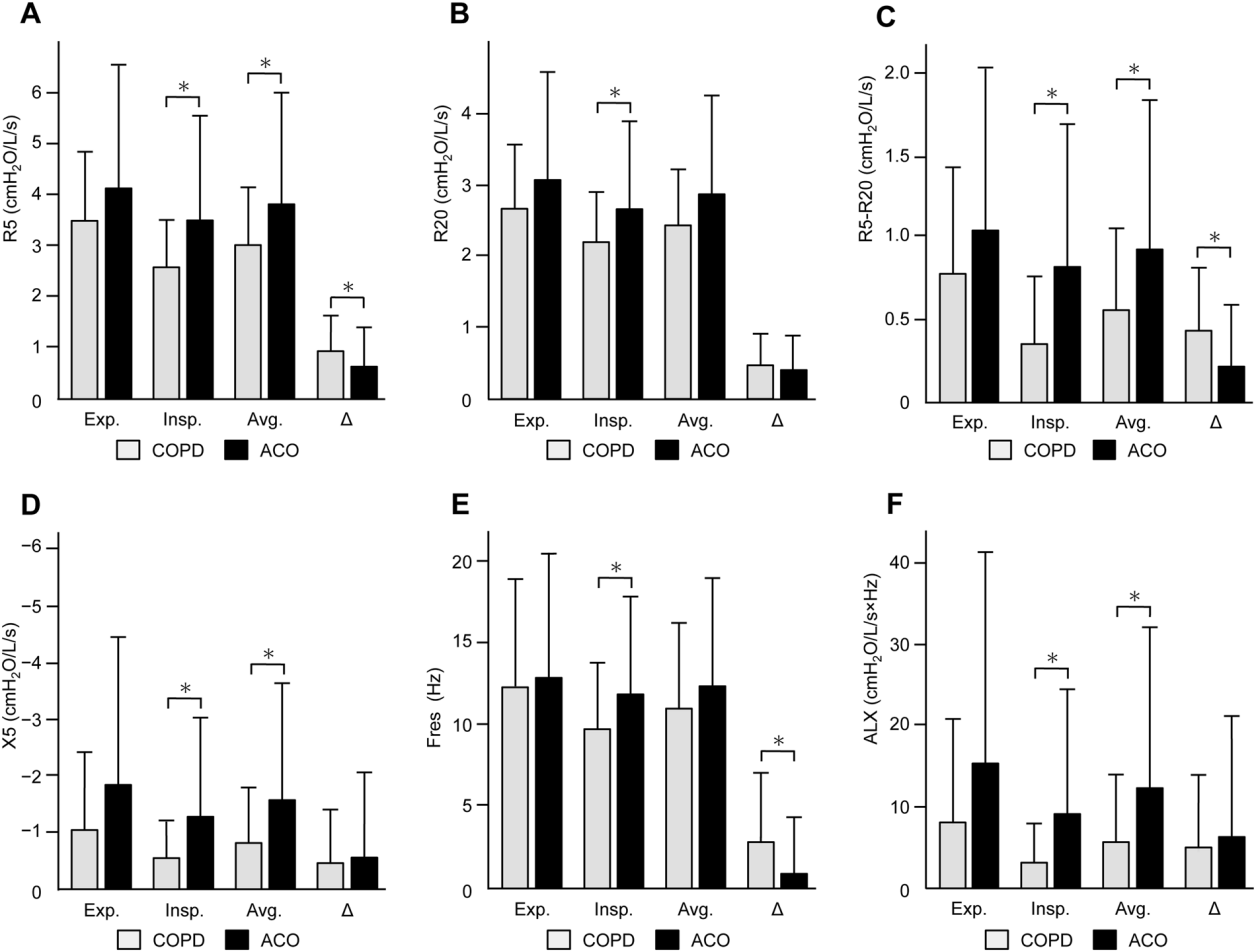
Comparison of respiratory resistance and reactance between COPD and ACO. (**A**) Respiratory resistance at 5 Hz (R5). (**B**) Respiratory resistance at 20 Hz (R20). (**C**) Difference between R5 and R20 (R5–R20). (**D**) Respiratory reactance at 5 Hz (X5). (**E**) Resonant frequency (Fres). (**F**) Low-frequency reactance area (ALX). COPD, chronic obstructive pulmonary disease; ACO, asthma–COPD overlap; exp., expiratory phase: insp., inspiratory phase; avg., average value of the inspiratory and expiratory phases; Δ, gap between the inspiratory and expiratory phases. Grey and black bars indicate COPD and ACO, respectively.

Patients with ACO showed consistently higher resistance and reactance during the inspiratory and expiratory phases (Fig. 2, Supplementary Table S6). In the inspiratory phase, respiratory resistance at 5 Hz (R5) and 20 Hz (R20), the difference between R5 and R20 (R5–R20), respiratory reactance at 5 Hz (X5), resonant frequency (Fres) and low-frequency reactance area (ALX) were significantly higher in patients with ACO than in matched patients with COPD (*p* = 0.006, *p* = 0.025, *p* = 0.002, *p* = 0.018, *p* = 0.028, and *p* = 0.021, respectively). At the average of the inspiratory and expiratory phases (avg.), R5_avg._, (R5−R20)_avg._, X5_avg._, and ALX_avg._ were significantly higher in patients with ACO than in matched patients with COPD (*p* = 0.031, *p* = 0.017, *p* = 0.035, and *p* = 0.047, respectively). With regard to the gap between the expiratory and inspiratory phases (Δ), patients with ACO had a significantly lower ΔR5, Δ(R5−R20), and ΔFres compared with matched patients with COPD (*p* = 0.036, *p* = 0.006, and *p* = 0.006, respectively). Variable clustering analysis selected R5_avg._, X5_avg_., and Δ(R5–R20) as representative variables of respiratory impedance indices (Supplementary Table S7). In multivariate logistic regression analysis, a higher R5_avg._ and lower Δ(R5–R20) were significant predictive factors for ACO (Table 3). A cut-off value for the diagnosis of ACO was 4.32 cm H_2_O/L/s in R5_avg._ (sensitivity, 0.33; specificity, 0.87; and AUC, 0.58) and 0.15 cm H_2_O/L/s in Δ(R5–R20) (sensitivity, 0.51; specificity, 0.81; and AUC, 0.65). When using the second most representative variables, a higher R5 at the expiratory phase (instead of R5_avg._) and lower ΔR5 (instead of Δ(R5–R20)) were selected as significant predictive factors for ACO (Supplementary Table S8). Univariate regression analyses for unselected respiratory impedance variables are shown in Supplementary Table S5.

**Table 3:** Logistic regression analyses of respiratory impedance for ACO.

Variables	Univariate		Multivariate	
	Odds ratio	*p*-value	Odds ratio	*p*-value
Age, per 1-year increase	0.99 (0.95–1.03)	0.638	0.99 (0.94–1.04)	0.695
Sex: female	2.59 (0.81–8.58)	0.107	0.84 (0.20–3.41)	0.806
R5_avg._, per 1 cm H_2_O/L/s increase	1.37 (1.08–1.79)	0.014	2.17 (1.28–3.88)	0.004
Δ(R5–R20), per 1 cm H_2_O/L/s increase	0.25 (0.08–0.67)	0.010	0.06 (0.01–0.28)	<0.001
X5_avg._, per 1 cm H_2_O/L/s increase	0.72 (0.54–0.92)	0.015	1.24 (0.73–2.11)	0.429

Data are expressed as odds ratios (95% confident intervals). R5, respiratory resistance at 5 Hz; R 20, respiratory resistance at 20 Hz; X5, respiratory reactance at 5 Hz; avg., average value of the inspiratory and expiratory phases; Δ, gap between the inspiratory and expiratory phases.

## Discussion

In the current study, we compared respiratory impedance and airway structural changes in well-balanced patients with ACO and COPD. Patients with ACO had (1) consistently higher respiratory resistance and reactance during tidal breathing and (2) a smaller gap between inspiratory and expiratory phases. With regard to 3D-CT analyses, patients with ACO had (1) a greater WT/√BSA from third- to fourth-generation bronchi, (2) a greater %WT from third- to sixth-generation bronchi, (3) a smaller Ai/BSA from fifth- to sixth-generation bronchi, and (4) less emphysematous changes. Diagnosis of asthma or COPD is usually based on subjective assessments of medical interviewing and conventional pulmonary function tests, including spirometry and peak expiratory flow measurements^1,2,4^. However, these assessments are often insufficient to distinguish between COPD and ACO. In our study, patients with COPD and ACO had a comparable smoking history and spirometry, and thus were not easily distinguishable by the conventional approach alone. Assessments using respiratory impedance and 3D-CT showed different physiological and morphological airway features in patients with COPD and ACO. This may help to understand the difference between these two diseases phenotypes.

Higher respiratory resistance in ACO suggests that ACO has greater airway narrowing than COPD, which is supported by our results of 3D-CT analyses of the airways. Respiratory resistance reflects the forward pressure of airways^14,15^, and is affected by airway calibre^16,23,24^. In our study, indices of respiratory resistance were correlated with Ai/BSA (Supplementary Table S9). Among indices of resistance, R5 denotes total airway resistance, while R20 indicates the central airway^14,16^. Our 3D-CT analyses in the third- to sixth-generation central bronchi showed that ACO had greater airway narrowing, which might explain the higher R20. Additionally, the higher R5 in ACO suggested that ACO had advanced airway narrowing in more distal bronchi beyond the measurement rage of 3D-CT analyses.

The higher respiratory reactance in ACO suggests that ACO has more severe small airway disease than COPD. Respiratory reactance indicates dysfunction of small airways^11,12,25^. Small airway dysfunction is a major pathogenesis in COPD^26,27^, which results in impaired respiratory reactance^11,12,17,28,29^. Asthma is also associated with small airway disease and impaired respiratory reactance^6,7,10,12,13^. However, the underlying mechanisms for small airway dysfunction in asthma might be different from those in COPD^30^. Few studies have assessed small airways in ACO or compared them with COPD. Therefore, the pathophysiological mechanisms involved in the difference in respiratory reactance between ACO and COPD are unknown.

A gap in respiratory impedance between the inspiratory and expiratory phases characterizes the pathophysiological differences between ACO and COPD. A marked increase in respiratory resistance and reactance in the expiratory phase and the resulting wide gap within the respiratory phases are important features of respiratory physiology in COPD^11,12,17,31^. A dynamic decrease in the airway lumen occurs during the expiratory phase in COPD^18,32^, which may be responsible for the large gap in respiratory impedance within the respiratory phase. This pathophysiological feature of COPD was significant, even when compared with ACO in our study. In contrast, patients with ACO had a higher respiratory resistance and reactance in the inspiratory phase and at the average of the two breathing phases compared with patients with COPD. This resulted in a smaller gap within the respiratory phases. This suggests that patients with ACO have consistent airway narrowing, even during the inspiratory phase. Images of 3D-CT at full inspiration and expansion of the airways showed that ACO had a smaller bronchial airway lumen compared with COPD. Evaluating respiratory impedance during the inspiratory and expiratory phases may be important for understanding the physiological differences between ACO and COPD.

Our 3D-CT analyses showed that ACO had greater morphological airway changes in central bronchi and less emphysematous changes than did COPD. The pathogenic features of the airways in asthma and COPD have been investigated separately. However, only a few reports have directly compered the structural differences of the airway between these two diseases. Hartley *et al*. reported that patients with COPD had more severe emphysema compared with those with asthma, but there was no significant difference in the mean bronchial lumen area, mean bronchial wall area, or percentage wall area, as assessed by CT^33^. However, in their study, patients with asthma and COPD showed differences in age, the proportion of women, smoking history, and airflow limitation assessed by spirometry. Shimizu *et al*. reported that patients with asthma had a greater percentage wall area in third- to fifth-generation bronchi, smaller Ai in third- to sixth-generation bronchi, and less low-attenuation volume of the lungs compared with patients with COPD who had comparable characteristics, including spirometric data^34^. They assumed that airway remodelling in asthma occurs in the proximal airways, whereas that in COPD occurs in the small airways. Hardin *et al*. reported that patients with ACO had a greater airway wall area percentage in segmental and subsegmental bronchi, and less emphysema compared with COPD without asthma, after adjustment for demographics in multivariate regression analyses^35^. These data, including our data, indicate that ACO has distinct morphological features from COPD and has some similarities to those in asthma. In addition, when adjusted for %LAA using logistic regression analyses (Supplementary Table S9), patients with ACO and COPD had differences in respiratory impedance and 3D-CT, similarly as observed in the original analysis (except that only ΔR5, ΔFres, and %WT in third-generation bronchi did not reach statistical significance), which suggested that physiological and morphological differences between ACO and COPD may be independent from emphysema.

The current study has limitations. First, only a few female patients were evaluated in this study because ACO and COPD are male-dominant characteristics. Women are thought to have a different sensitivity to smoking compared with men^36^. Therefore, sex differences in ACO should be further investigated. Second, the observed airways were limited to central third- to six-generation bronchi because of the resolution limits of CT. The morphological and pathological differences of the small airways between ACO and COPD are still unknown. Third, inhaler therapies were unmatched between the two groups because of the small sample size. It has been reported that combination therapy with ICS and LABA increases Ai and decreases WT in patients with COPD^37^. In the present study, patients with ACO received ICS and LABA more frequently. Therefore, patients with ACO may demonstrate greater reductions in Ai and greater increases in WT if they received less ICS and LABA. The direct influence of inhaler therapies upon physiological and morphological changes merits further investigation. Fourth, there is still controversy and no universal definition for ACO including necessity and definition of eosinophilic inflammation, and further studies are required to understand this condition. When we employed other criteria recommended in a global expert panel discussion^38^ in this study population, 29 patients were defined as having ACO. The patients with the alternative definition of ACO also demonstrated the similar results, as observed in those with the initial definition of ACO (Supplementary Tables S10 and S11). Patients with both features of asthma and COPD (patients with ACO in this study) have greater symptoms, physical impairment, and more hospital admissions compared with asthma or COPD alone^39^. The term ACO should be used as an interim clinical label to identify at-risk patients on the basis of clinical features of asthma and COPD to safely manage patients until evidence on mechanisms and targeted treatments emerges. Our results may help understanding of the mechanisms of ACO and COPD.

In conclusion, patients with ACO have higher respiratory resistance and reactance during tidal breathing, and a smaller gap between the inspiratory and expiratory phases compared with patients with COPD. In 3D-CT analyses, patients with ACO have a greater bronchial wall thickness, a smaller bronchial lumen, and less emphysema compared with patients with COPD. The physiological and morphological differences between ACO and COPD might help to understand and distinguish these two diseases.

## Methods

### Subjects

Patients with COPD who satisfied the definition of COPD by the GOLD^1^ prospectively underwent measurements of respiratory impedance, spirometry, and a chest CT scan. The main inclusion criteria were patients aged ≥40 years old, those who had a smoking history ≥10 pack-years, and persistent airflow limitation defined as a ratio of forced expiratory volume in 1 second (FEV_1_)/forced vital capacity <70%. The patients were required to be clinically stable, which was defined as no requirement of treatment change, no respiratory tract infection, and no exacerbation within 4 weeks before enrolment into the study. Patients who received long-term oxygen therapy and those who had diffuse lung diseases, neuromuscular diseases, congenital anomalies of the bronchial tree, or a history of thoracic surgery were also excluded. Among these patients with COPD, those who had (1) a history of variable respiratory symptoms (wheeze, shortness of breath, chest tightness, or cough) and (2) variable expiratory airflow limitation (increase in percentage predicted FEV_1_ of >12% and FEV_1_ of >200 mL after a postbronchodilator or 4 weeks of anti-inflammatory treatment) were defined as having ACO^1,4^. We compared patients with ACO and the remaining patients with COPD without features of asthma.

This study was conducted in accordance with the ethical standards of the Declaration of Helsinki. The study protocol was approved by the Institutional Review Board of Hamamatsu University School of Medicine (Hamamatsu, Japan, approval No. 25-182). Each patient provided written informed consent for inclusion in the study.

### Analysis of 3D-CT

The detailed method of 3D-CT analysis of the airways has been described elsewhere^23,24,40^. In brief, multiple-detector-row CT (MDCT) imaging was performed using a 64-slice MDCT machine (Aquilion-64; Toshiba Medical Systems, Tokyo, Japan) in the supine position at full inspiration breath-hold. In 3D-reconstructed bronchial images obtained from MDCT data using SYNAPSE VINCENT (Fuji Film, Tokyo, Japan), WT (mm), %WT to the outer diameter of the airway, and Ai (mm^2^) were automatically computed. Measurements of WT and Ai were performed in the midpoint of four levels of bronchi (third-generation [segmental], fourth-generation [sub-segmental], fifth-generation, and sixth-generation bronchi) in six airways that originated from six segmental bronchi (B1, B2, B3, B8, B9, and B10) in the right lung. The Ai and WT for each generation of bronchi were expressed as the mean of six airways and adjusted by BSA to eliminate the potential effect of body size of each patient. The %LAA, defined as the percentage of area below −950 HU in the total lung area, was calculated using SYNAPSE VINCENT.

### Measurement of physiological function

Detailed information on spirometry and measurements of respiratory impedance were previously described^23,24^. Briefly, spirometry was performed using Autospirometer System 7 (Minato Medical Science, Osaka, Japan) according to standards set by the Japanese Respiratory Society^41^. Respiratory impedance was measured using a forced oscillation technique device (Most-Graph 01; Chest MI, Tokyo, Japan) according to standard recommendations, as reported previously^12,15^. Both physiological function tests were performed on the same day as CT of the chest. The measurement of respiratory impedance was performed before spirometry to avoid the effect of forced breathing. Short-acting β_2_-agonists were not used for >12 h before these tests. We evaluated the following: R5, R20, R5–R20, X5 (which had a negative value for higher reactance), Fres (where the reactance crosses zero and the elastic and inertial forces are equal in magnitude and opposite), and ALX (the integral of reactance from 5 Hz to Fres). Each index was measured during tidal breathing and was separately expressed as the inspiratory phase, expiratory phase, avg., and Δ.

### Statistical analyses

Welch’s t test was used to analyse continuous variables and Fisher’s exact test was used for categorical groups. Correlations between 3D-CT parameters and indices of respiratory impedance were evaluated using Pearson’s correlation coefficient. For propensity score-matched analysis, a logistic regression analysis was undertaken using age, sex, BMI, and pack-year smoking to calculate propensity scores. Then, one-to-two optimal matching was undertaken to minimize the sum of difference in propensity scores between two groups. To select variables for multivariate logistic regression analysis, variable clustering analysis was used for 3D-CT parameters and respiratory impedance indices with a *p* value of <0.1 in univariate logistic regression analysis. The squared correlation of a variable with its cluster component (R^2^ with Own) and that with its next closest cluster (R^2^ with next closest) were separately calculated. A variable with a minimum ratio of (1−R^2^ with Own)/(1−R^2^ with next closest) in a cluster was selected as the representative variable of the cluster. A cut-off value for the receiver operating characteristic curve was determined by the Youden index, defined as a maximum value of (sensitivity + specificity − 1). A *p* value < 0.05 (two-sided) was considered significant. All values were analysed using JMP v13.0.0 (SAS Institute Japan, Tokyo, Japan), except for propensity score matching using EZR (Saitama Medical Centre, Jichi Medical University, Saitama, Japan). EZR is a graphical user interface for R (The R Foundation for Statistical Computing, Vienna, Austria).

## Supplementary information

Supplementary Table
