# Supplementary material for: Physiological and morphological differences of airways between COPD and asthma–COPD overlap
Source: Sci Rep. 2019 May 24;9:7818. doi: 10.1038/s41598-019-44345-6 (PMC6534606; doi:10.1038/s41598-019-44345-6)
Supplement: Supplementary file 1 — Supplementary Table [file 41598_2019_44345_MOESM1_ESM.docx]

**Physiological and morphological differences of airways between COPD and asthma–COPD overlap**

Masato Karayama^1^, Naoki Inui^1,2^*, Hideki Yasui^1^, Masato Kono^1^, Hironao Hozumi^1^, Yuzo Suzuki^1^, Kazuki Furuhashi^1^, Dai Hashimoto^1^, Noriyuki Enomoto^1^, Tomoyuki Fujisawa^1^, Yutaro Nakamura^1^, Hiroshi Watanabe^2^, Takafumi Suda^1^

^1^Second Division, Department of Internal Medicine, Hamamatsu University School of Medicine, 1-20-1 Handayama, Hamamatsu 431-3192, Japan

^2^Department of Clinical Pharmacology and Therapeutics, Hamamatsu University School of Medicine, 1-20-1 Handayama, Hamamatsu 431-3192, Japan

**Corresponding author:** Naoki Inui MD, PhD

Hamamatsu University School of Medicine

1-20-1 Handayama, Hamamatsu 431-3192, Japan

Tel: +81-53-435-2263

Fax: +81-53-435-2386

E-mail: [inui@hama-med.ac.jp](mailto:inui@hama-med.ac.jp)

**Supplementary Table S1: Patient characteristics of unmatched population.**

|  | ACO (n=43) | Unmatched COPD (n=124) |
| --- | --- | --- |
| Age, years | 69.3 (7.7) | 71.5 (8.9) |
| Sex: male | 36 (83.7) | 117 (94.4) |
| Smoking status  Current smoker  Former smoker  Pack-year | 5 (11.6)  38 (88.4)  39.8 (37.2) | 28 (22.6)  96 (77.4)  59.0 (38.8)* |
| BMI (kg/m^2^) | 24.2 (4.6) | 22.5 (3.4)** |
| Pulmonary function tests  FVC, % predicted  FEV_1_, % predicted  FEV_1_/FVC (%)  FEF_25-75_, % predicted | 91.8 (13.7)  69.4 (19.0)  60.6 (13.9)  34.3 (18.8) | 93.9 (17.5)  67.6 (21.3)  56.6 (12.3)  32.0 (17.6) |
| GOLD stage  I  II  III  IV | 12 (27.9)  24 (55.8)  4 (9.3)  3 (7.0) | 36 (29.0)  62 (50.0)  19 (15.3)  7 (5.7) |
| Treatment  ICS  LABA  LAMA | 36 (83.7)  31 (72.1)  11 (25.6) | 8 (6.5)*  66 (53.2)**  75 (60.5)* |

Data are expressed as number (%) or mean (standard deviation). COPD, chronic obstructive pulmonary disease; ACO, asthma-COPD overlap; BMI, body mass index; FVC, forced vital capacity; FEV_1_, forced expiratory volume in 1 second; FEF, forced expiratory flow rate; GOLD, Global Initiative for Chronic Obstructive Lung Disease; ICS, inhaled corticosteroid; LABA, long-acting beta 2 agonist; LAMA, long-acting muscarinic antagonist, ^§^Data are expressed as fluticasone equivalent among patients receiving inhaled corticosteroid. **p*<0.001 compared with ACO. ***p*<0.05 compared with ACO.

**Supplementary Table S2. Three-dimensional CT imaging of the lungs**

|  | ACO (n=43) | Matched COPD (n=86) | *p*-value |
| --- | --- | --- | --- |
| Ai/BSA (mm^2^/mm^2^) |  |  |  |
| 3rd-generation bronchi | 20.75 (8.10) | 22.96 (6.54) | 0.127 |
| 4th-generation bronchi | 5.74 (2.05) | 6.27 (1.98) | 0.165 |
| 5th-generation bronchi | 3.14 (1.20) | 3.68 (1.21) | 0.018 |
| 6th-generation bronchi | 1.67 (0.78) | 2.11 (0.84) | 0.004 |
| WT/√BSA (mm/mm) |  |  |  |
| 3rd-generation bronchi | 1.29 (0.12) | 1.22 (0.13) | 0.003 |
| 4th-generation bronchi | 1.12 (0.09) | 1.07 (0.10) | 0.001 |
| 5th-generation bronchi | 0.98 (0.07) | 0.95 (0.09) | 0.075 |
| 6th-generation bronchi | 0.86 (0.08) | 0.86 (0.09) | 0.584 |
| %WT (%) |  |  |  |
| 3rd-generation bronchi | 35.5 (5.3) | 33.4 (3.6) | 0.023 |
| 4th-generation bronchi | 39.6 (5.0) | 37.3 (4.5) | 0.015 |
| 5th-generation bronchi | 43.2 (4.6) | 41.0 (5.6) | 0.017 |
| 6th-generation bronchi | 46.6 (5.2) | 44.5 (4.9) | 0.033 |
| %LAA | 31.6 (8.3) | 38.3 (9.7) | <0.001 |

Data are expressed as mean (standard deviation). Ai, airway inner luminal area; WT, airway wall thickness; %WT, percentage of airway wall thickness; %LAA, percentage of low attenuation area < −950 HU; BSA, body surface area.

**Supplementary Table S3. Variable clustering analysis of 3D-CT parameters**

| Cluster | Variables | R^2^ with own cluster | R^2^ with next closest cluster | Ratio of [1−R^2^] |
| --- | --- | --- | --- | --- |
| I | 5th-Ai/BSA | 0.831 | 0.085 | 0.185 |
|  | 6th-Ai/BSA | 0.755 | 0.124 | 0.280 |
|  | 3rd-%WT | 0.665 | 0.289 | 0.470 |
|  | 4th-%WT | 0.800 | 0.293 | 0.283 |
|  | 5th-%WT | 0.804 | 0.318 | 0.287 |
|  | 6th-%WT | 0.546 | 0.236 | 0.595 |
| II | 3rd-WT/√BSA | 0.914 | 0.261 | 0.117 |
|  | 4th-WT/√BSA | 0.914 | 0.264 | 0.117 |
| III | %LAA | 1 | 0.006 | 0 |

R^2^, the squared correlation of a variable; Ai, airway inner luminal area; WT, airway wall thickness; %WT, percentage of airway wall thickness; %LAA, percentage of low attenuation area < −950 HU; BSA, body surface area.

**Supplementary Table S4. Multivariate logistic regression analyses of 3D-CT for ACO using the second representative factors**

|  | Set 1 | | Set 2 | | Set 3 | |
| --- | --- | --- | --- | --- | --- | --- |
| Variables | Odds ratio | *p*-value | Odds ratio | *p*-value | Odds ratio | *p*-value |
| Age, per 1-year increase | 0.99 (0.94 − 1.04) | 0.749 | 0.99 (0.94 − 1.04) | 0.608 | 0.99 (0.94 − 1.04) | 0.699 |
| Sex: female | 2.38 (0.58 − 10.45) | 0.228 | 2.30 (0.56 − 9.94) | 0.248 | 2.25 (0.53 − 10.05) | 0.270 |
| 3rd-WT/√BSA, per 0.1mm/mm increase | 1.51 (1.06 − 2.20) | 0.022 |  |  |  |  |
| 4th-WT/√BSA^§^, per 0.1mm/mm increase |  |  | 1.99 (1.25 − 3.33) | 0.003 | 1.78 (1.10 − 3.02) | 0.019 |
| 5th-Ai/BSA, per 1mm^2^/mm^2^ increase |  |  | 0.78 (0.53 − 1.12) | 0.175 |  |  |
| 6th-Ai/BSA^§^, per 1mm^2^/mm^2^ increase | 0.52 (0.27 − 0.93) | 0.027 |  |  | 0.55 (0.28 − 0.99) | 0.046 |
| %LAA, per 1% increase | 0.91 (0.87 − 0.96) | <0.001 | 0.92 (0.87 − 0.96) | <0.001 | 0.91 (0.86 − 0.96) | <0.001 |

Data are expressed as odds ratio (95% confident interval). Ai, airway inner luminal area; WT, airway wall thickness; %LAA, percentage of low attenuation area < −950 HU; BSA, body surface area. ^§^The second representative factors at valuable clustering analyses. Set 1, 2, and 3 includes different combinations of the first and second representative factors in the valuable clustering analyses.

**Supplementary Table S5. Univariate logistic regression analyses of respiratory impedance and 3D-CT for ACO**

| Variables | Odds ratio | *p*-value |
| --- | --- | --- |
| Age, per 1-year increase | 0.99 (0.95 − 1.03) | 0.638 |
| Sex: female | 2.59 (0.81 – 8.58) | 0.107 |
| 3rd-Ai/BSA, per 1mm^2^/ mm^2^ increase | 0.96 (0.90 − 1.01) | 0.103 |
| 4th-Ai/BSA, per 1mm^2^/ mm^2^ increase | 0.87 (0.71 − 1.05) | 0.160 |
| 5th-Ai/BSA, per 1mm^2^/ mm^2^ increase | 0.68 (0.48 – 0.93) | 0.020 |
| 6th-Ai/BSA, per 1mm^2^/ mm^2^ increase | 0.49 (0.28 – 0.80) | 0.007 |
| 3rd-WT/√BSA, per 0.1mm/mm increase | 1.56 (1.16 – 2.15) | 0.005 |
| 4th-WT/√BSA, per 0.1mm/mm increase | 1.84 (1.24 – 2.83) | 0.004 |
| 5th-WT/√BSA, per 0.1mm/mm increase | 1.42 (0.94 – 2.20) | 0.103 |
| 6th-WT/√BSA, per 0.1mm/mm increase | 1.13 (0.72 – 1.75) | 0.597 |
| 3rd-%WT, per 1% increase | 1.12 (1.03 – 1.23) | 0.013 |
| 4th-%WT, per 1% increase | 1.11 (1.03 – 1.22) | 0.014 |
| 5th-%WT, per 1% increase | 1.09 (1.01 – 1.18) | 0.029 |
| 6th-%WT, per 1% increase | 1.09 (1.01 – 1.17) | 0.032 |
| %LAA, per 1% increase | 0.92 (0.88 − 0.96) | <0.001 |
| R5_exp._, per 1cmH_2_O/L/s increase | 1.22 (0.99 − 1.52) | 0.066 |
| R5_insp._, per 1cmH_2_O/L/s increase | 1.60 (1.21 – 2.20) | 0.002 |
| ΔR5, per 1cmH_2_O/L/s increase | 0.54 (0.28 – 0.93) | 0.038 |
| R5_avg._, per 1cmH_2_O/L/s increase | 1.37 (1.08 – 1.79) | 0.014 |
| R20_exp._, per 1cmH_2_O/L/s increase | 1.33 (0.97 − 1.90) | 0.086 |
| R20_insp._, per 1cmH_2_O/L/s increase | 1.71 (1.15 – 2.67) | 0.012 |
| ΔR20, per 1cmH_2_O/L/s increase | 0.65 (0.27 – 1.47) | 0.321 |
| R20_avg._, per 1cmH_2_O/L/s increase | 1.51 (1.05 − 2.26) | 0.035 |
| (R5-R20)_exp._, per 1cmH_2_O/L/s increase | 1.53 (0.96 − 2.51) | 0.076 |
| (R5-R20)_insp._, per 1cmH_2_O/L/s increase | 3.62 (1.83 – 8.21) | <0.001 |
| Δ(R5-R20), per 1cmH_2_O/L/s increase | 0.25 (0.08 − 0.67) | 0.010 |
| (R5-R20)_avg._, per 1cmH_2_O/L/s increase | 2.23 (1.28 – 4.18) | 0.007 |
| X5_exp._, per 1cmH_2_O/L/s increase | 0.81 (0.67 – 0.98) | 0.036 |
| X5_insp._, per 1cmH_2_O/L/s increase | 0.52 (0.31 – 0.79) | 0.006 |
| ΔX5, per 1cmH_2_O/L/s increase | 0.93 (0.68 − 1.29) | 0.647 |
| X5_avg._, per 1cmH_2_O/L/s increase | 0.72 (0.54 – 0.92) | 0.015 |
| Fres_exp._, per 1Hz increase | 1.01 (0.96 − 1.07) | 0.638 |
| Fres_insp._, per 1Hz increase | 1.10 (1.02 – 1.19) | 0.017 |
| ΔFres, per 1Hz increase | 0.85 (0.74 – 0.96) | 0.014 |
| Fres_avg._, per 1Hz increase | 1.04 (0.98 − 1.11) | 0.183 |
| ALX_exp._, per 1cmH_2_O/L/s×Hz increase | 1.02 (1.01 – 1.04) | 0.045 |
| ALX_insp._, per 1cmH_2_O/L/s×Hz increase | 1.07 (1.02 – 1.14) | 0.010 |
| ΔALX, per 1cmH_2_O/L/s×Hz increase | 1.01 (0.98 − 1.04) | 0.501 |
| ALX_avg._, per 1cmH_2_O/L/s×Hz increase | 1.03 (1.01 – 1.07) | 0.020 |

Data are expressed as odds ratio (95% confident interval). Ai, airway inner luminal area; WT, airway wall thickness; %WT, percentage of airway wall thickness; %LAA, percentage of low attenuation area < −950 HU; BSA, body surface area; R5, respiratory resistance at 5 Hz; R 20, respiratory resistance at 20 Hz: X5, respiratory reactance at 5 Hz; Fres, resonant frequency; ALX, low-frequency reactance area; exp, expiratory phase: insp, inspiratory phase; avg, average of inspiratory and expiratory phase; Δ, gap between inspiratory and expiratory phase.

**Supplementary Table S6. Respiratory impedance**

|  | ACO (n=43) | Matched COPD (n=86) | *p*-value |
| --- | --- | --- | --- |
| Expiratory phase | | | |
| R5 (cmH_2_O/L/s) | 4.12 (2.44) | 3.47 (1.38) | 0.111 |
| R20 (cmH_2_O/L/s) | 3.07 (1.54) | 2.68 (0.90) | 0.134 |
| R5-R20 (cmH_2_O/L/s) | 1.05 (0.98) | 0.79 (0.64) | 0.116 |
| X5 (cmH_2_O/L/s) | −1.84 (2.62) | −1.06 (1.36) | 0.074 |
| Fres (Hz) | 12.78 (7.70) | 12.17 (6.64) | 0.658 |
| ALX (cmH_2_O/L/s×Hz) | 15.19 (25.48) | 8.11 (12.33) | 0.091 |
| Inspiratory phase | | | |
| R5 (cmH_2_O/L/s) | 3.49 (2.05) | 2.55 (0.96) | 0.006 |
| R20 (cmH_2_O/L/s) | 2.67 (1.24) | 2.20 (0.72) | 0.025 |
| R5-R20 (cmH_2_O/L/s) | 0.82 (0.87) | 0.35 (0.41) | 0.002 |
| X5 (cmH_2_O/L/s) | −1.26 (1.75) | −0.58 (0.63) | 0.018 |
| Fres (Hz) | 11.93 (5.95) | 9.65 (4.20) | 0.028 |
| ALX (cmH_2_O/L/s×Hz) | 8.91 (15.22) | 3.22 (4.80) | 0.021 |
| Average of inspiratory and expiratory phase | | | |
| R5 (cmH_2_O/L/s) | 3.81 (2.22) | 3.01 (1.13) | 0.031 |
| R20 (cmH_2_O/L/s) | 2.87 (1.38) | 2.44 (0.78) | 0.063 |
| R5-R20 (cmH_2_O/L/s) | 0.94 (0.91) | 0.57 (0.50) | 0.017 |
| X5 (cmH_2_O/L/s) | −1.55 (2.10) | −0.82 (0.95) | 0.035 |
| Fres (Hz) | 12.36 (6.73) | 10.91 (5.24) | 0.221 |
| ALX (cmH_2_O/L/s×Hz) | 12.05 (19.68) | 5.66 (8.25) | 0.047 |
| Gap between inspiratory and expiratory phase | | | |
| R5 (cmH_2_O/L/s) | 0.63 (0.74) | 0.92 (0.71) | 0.036 |
| R20 (cmH_2_O/L/s) | 0.40 (0.51) | 0.49 (0.44) | 0.346 |
| R5-R20 (cmH_2_O/L/s) | 0.23 (0.39) | 0.44 (0.41) | 0.006 |
| X5 (cmH_2_O/L/s) | −0.57 (1.48) | −0.47 (0.94) | 0.695 |
| Fres (Hz) | 0.85 (2.89) | 2.52 (3.67) | 0.006 |
| ALX (cmH_2_O/L/s×Hz) | 6.28 (14.59) | 4.89 (8.82) | 0.567 |

Data are expressed as mean (standard deviation). ACO, asthma-COPD overlap; COPD, chronic obstructive pulmonary disease; R5, respiratory resistance at 5 Hz; R 20, respiratory resistance at 20 Hz: X5, respiratory reactance at 5 Hz; Fres, resonant frequency; ALX, low-frequency reactance area.

**Supplementary Table S7. Variable clustering analysis of respiratory impedance indices**

| Cluster | Variables | R^2^ with own cluster | R^2^ with next closest cluster | Ratio of [1−R^2^] |
| --- | --- | --- | --- | --- |
| I | R5_exp._ | 0.974 | 0.533 | 0.057 |
|  | R5_insp._ | 0.945 | 0.692 | 0.178 |
|  | R5_avg._ | 0.999 | 0.626 | 0.002 |
|  | R20_exp._ | 0.897 | 0.440 | 0.184 |
|  | R20_insp._ | 0.890 | 0.503 | 0.221 |
|  | R20_avg._ | 0.929 | 0.487 | 0.138 |
|  | (R5-R20)_exp._ | 0.807 | 0.519 | 0.401 |
|  | (R5-R20)_avg._ | 0.823 | 0.667 | 0.530 |
| II | ΔR5 | 0.836 | 0.245 | 0.217 |
|  | Δ(R5-R20) | 0.850 | 0.150 | 0.177 |
|  | ΔFres | 0.667 | 0.160 | 0.397 |
| III | (R5-R20)_insp._ | 0.746 | 0.706 | 0.867 |
|  | X5_exp._ | 0.921 | 0.507 | 0.160 |
|  | X5_insp._ | 0.858 | 0.571 | 0.331 |
|  | X5_avg._ | 0.976 | 0.578 | 0.056 |
|  | Fres_insp._ | 0.789 | 0.432 | 0.371 |
|  | ALX_exp._ | 0.905 | 0.480 | 0.183 |
|  | ALX_insp._ | 0.896 | 0.537 | 0.225 |
|  | ALX_avg._ | 0.972 | 0.539 | 0.061 |

R^2^, the squared correlation of a variable; R5, respiratory resistance at 5 Hz; R 20, respiratory resistance at 20 Hz: X5, respiratory reactance at 5 Hz; Fres, resonant frequency; ALX, low-frequency reactance area; exp, expiratory phase: insp, inspiratory phase; avg, average of inspiratory and expiratory phase; Δ, gap between inspiratory and expiratory phase.

**Supplementary Table S8. Multivariate logistic regression analyses of respiratory impedance for ACO using the second representative factors**

|  | Set 1 | | Set 2 | |
| --- | --- | --- | --- | --- |
| Variables | Odds ratio | *p*-value | Odds ratio | *p*-value |
| Age, per 1-year increase | 0.99 (0.94 − 1.04) | 0.659 | 0.98 (0.94 − 1.03) | 0.530 |
| Sex: female | 0.89 (0.21 − 3.68) | 0.874 | 1.55 (0.42 − 5.71) | 0.506 |
| R5_exp._^§^, per 1cmH_2_O/L/s increase | 1.89 (1.14 − 3.25) | 0.013 | 2.07 (1.25 − 3.62) | 0.005 |
| ΔR5^§^, per 1cmH_2_O/L/s increase |  |  | 0.13 (0.04 − 0.38) | <0.001 |
| Δ(R5-R20), per 1cmH_2_O/L/s increase | 0.05 (0.01 – 0.27) | <0.001 |  |  |
| X5_avg._, per 1cmH_2_O/L/s increase | 1.12 (0.67 − 1.89) | 0.658 | 1.09 (0.67 − 1.80) | 0.736 |
|  | Set 3 | | Set 4 | |
| Variables | Odds ratio | *p*-value | Odds ratio | *p*-value |
| Age, per 1-year increase | 0.98 (0.94 − 1.03) | 0.530 | 0.99 (0.94 − 1.04) | 0.572 |
| Sex: female | 1.55 (0.42 − 5.71) | 0.506 | 1.47 (0.39 − 5.51) | 0.559 |
| R5_avg._, per 1 cmH_2_O/L/s increase | 2.07 (1.25 − 3.62) | 0.005 | 2.22 (1.33 − 3.92) | 0.002 |
| ΔR5^§^, per 1 cmH_2_O/L/s increase | 0.19 (0.06 − 0.46) | <0.001 | 0.18 (0.06 − 0.44) | <0.001 |
| X5_avg._, per 1 cmH_2_O/L/s increase | 1.09 (0.67 − 1.80) | 0.735 |  |  |
| ALX_avg._^§^, per 1 cmH_2_O/L/s×Hz increase |  |  | 0.98 (0.93 - 1.03) | 0.492 |
|  | Set 5 | | Set 6 | |
| Variables | Odds ratio | *p*-value | Odds ratio | *p*-value |
| Age, per 1-year increase | 0.99 (0.94 − 1.04) | 0.572 | 0.99 (0.94 − 1.04) | 0.636 |
| Sex: female | 1.48 (0.39 − 5.51) | 0.559 | 0.88 (0.20 − 3.67) | 0.865 |
| R5_exp._^§^, per 1 cmH_2_O/L/s increase | 2.22 (1.33 − 3.91) | 0.002 | 1.88 (1.17 − 3.17) | 0.009 |
| ΔR5^§^, per 1 cmH_2_O/L/s increase | 0.12 (0.03 − 0.35) | <0.001 |  |  |
| Δ(R5-R20), per 1 cmH_2_O/L/s increase |  |  | 0.05 (0.01 − 0.27) | <0.001 |
| ALX_avg._^§^, per 1 cmH_2_O/L/s×Hz increase | 0.98 (0.93 − 1.03) | 0.493 | 0.99 (0.94 − 1.04) | 0.648 |
|  | Set 7 | |  |  |
| Variables | Odds ratio | *p*-value |  |  |
| Age, per 1-year increase | 0.99 (0.94 − 1.04) | 0.663 |  |  |
| Sex: female | 0.82 (0.19 − 3.37) | 0.779 |  |  |
| R5_avg._, per 1 cmH_2_O/L/s increase | 2.17 (1.30 − 3.82) | 0.003 |  |  |
| Δ(R5-R20), per 1 cmH_2_O/L/s increase | 0.06 (0.01 − 0.27) | <0.001 |  |  |
| ALX_avg._^§^, per 1 cmH_2_O/L/s×Hz increase | 0.98 (0.93 - 1.03) | 0.406 |  |  |

Data are expressed as odds ratio (95% confident interval). R5, respiratory resistance at 5 Hz; R 20, respiratory resistance at 20 Hz: X5, respiratory reactance at 5 Hz; ALX, low-frequency reactance area; exp, expiratory phase: avg, an average of two breathing phase; Δ, gap between inspiratory and expiratory phase. ^§^Second representative factors at valuable clustering analyses. Set 1, 2, 3, 4, 5, 6, and 7 includes different combinations of first and second representative factors in the valuable clustering analyses.

**Supplementary Table S9. Logistic regression analyses of respiratory impedance and 3D-CT for ACO, adjusted by %LAA.**

| Variables | Odds ratio | *p*-value |
| --- | --- | --- |
| Age, per 1-year increase | 0.99 (0.95 − 1.04) | 0.951 |
| Sex: female | 2.41 (0.67 – 8.97) | 0.174 |
| 3rd-Ai/BSA, per 1mm^2^/ mm^2^ increase | 0.98 (0.92 − 1.04) | 0.491 |
| 4th-Ai/BSA, per 1mm^2^/ mm^2^ increase | 0.90 (0.73 − 1.10) | 0.330 |
| 5th-Ai/BSA, per 1mm^2^/ mm^2^ increase | 0.66 (0.46 – 0.93) | 0.021 |
| 6th-Ai/BSA, per 1mm^2^/ mm^2^ increase | 0.42 (0.22 – 0.73) | 0.004 |
| 3rd-WT/√BSA, per 0.1mm/mm increase | 1.64 (1.19 – 2.34) | 0.004 |
| 4th-WT/√BSA, per 0.1mm/mm increase | 2.08 (1.35 – 3.36) | 0.002 |
| 5th-WT/√BSA, per 0.1mm/mm increase | 1.48 (0.93 – 2.38) | 0.101 |
| 6th-WT/√BSA, per 0.1mm/mm increase | 1.18 (0.73 – 1.91) | 0.507 |
| 3rd-%WT, per 1% increase | 1.08 (0.98 – 1.19) | 0.102 |
| 4th-%WT, per 1% increase | 1.11 (1.02 – 1.21) | 0.024 |
| 5th-%WT, per 1% increase | 1.10 (1.01 – 1.19) | 0.026 |
| 6th-%WT, per 1% increase | 1.12 (1.03 – 1.23) | 0.009 |
| R5_exp._, per 1cmH_2_O/L/s increase | 1.26 (1.02 − 1.59) | 0.039 |
| R5_insp._, per 1cmH_2_O/L/s increase | 1.63 (1.22 – 2.29) | 0.002 |
| ΔR5, per 1cmH_2_O/L/s increase | 0.59 (0.31 – 0.107) | 0.101 |
| R5_avg._, per 1cmH_2_O/L/s increase | 1.41 (1.10 – 1.88) | 0.011 |
| R20_exp._, per 1cmH_2_O/L/s increase | 1.36 (0.97 − 1.93) | 0.072 |
| R20_insp._, per 1cmH_2_O/L/s increase | 1.72 (1.15 – 2.71) | 0.012 |
| ΔR20, per 1cmH_2_O/L/s increase | 0.67 (0.25 – 1.63) | 0.400 |
| R20_avg._, per 1cmH_2_O/L/s increase | 1.52 (1.05 − 2.29) | 0.032 |
| (R5-R20)_exp._, per 1cmH_2_O/L/s increase | 1.82 (1.09 − 3.19) | 0.026 |
| (R5-R20)_insp._, per 1cmH_2_O/L/s increase | 4.12 (1.98 – 9.97) | <0.001 |
| Δ(R5-R20), per 1cmH_2_O/L/s increase | 0.33 (0.10 − 0.95) | 0.050 |
| (R5-R20)_avg._, per 1cmH_2_O/L/s increase | 2.69 (1.45 – 5.52) | 0.004 |
| X5_exp._, per 1cmH_2_O/L/s increase | 0.74 (0.58 – 0.92) | 0.009 |
| X5_insp._, per 1cmH_2_O/L/s increase | 0.45 (0.25 – 0.72) | 0.003 |
| ΔX5, per 1cmH_2_O/L/s increase | 0.86 (0.60 − 1.23) | 0.400 |
| X5_avg._, per 1cmH_2_O/L/s increase | 0.63 (0.45 – 0.85) | 0.004 |
| Fres_exp._, per 1Hz increase | 1.04 (0.98 − 1.10) | 0.212 |
| Fres_insp._, per 1Hz increase | 1.13 (1.04 – 1.23) | 0.004 |
| ΔFres, per 1Hz increase | 0.88 (0.76 – 1.00) | 0.070 |
| Fres_avg._, per 1Hz increase | 1.08 (1.01 – 1.16) | 0.043 |
| ALX_exp._, per 1cmH_2_O/L/s×Hz increase | 1.03 (1.01 – 1.06) | 0.008 |
| ALX_insp._, per 1cmH_2_O/L/s×Hz increase | 1.09 (1.04 – 1.17) | 0.003 |
| ΔALX, per 1cmH_2_O/L/s×Hz increase | 1.02 (0.98 − 1.07) | 0.203 |
| ALX_avg._, per 1cmH_2_O/L/s×Hz increase | 1.05 (1.02 – 1.09) | 0.004 |

Each variable was evaluated in multivariate logistic regression analysis using %LAA as co-variable. Data are expressed as odds ratio (95% confident interval). Ai, airway inner luminal area; WT, airway wall thickness; %WT, percentage of airway wall thickness; %LAA, percentage of low attenuation area < −950 HU; BSA, body surface area; R5, respiratory resistance at 5 Hz; R 20, respiratory resistance at 20 Hz: X5, respiratory reactance at 5 Hz; Fres, resonant frequency; ALX, low-frequency reactance area; exp, expiratory phase: insp, inspiratory phase; avg, average of inspiratory and expiratory phase; Δ, gap between inspiratory and expiratory phase.

**Supplementary Table S10. Comparison of three-dimensional CT imaging of the lungs between ACO using different criteria and COPD**

|  | ACO using different criteria^#^ (n=29) | Matched COPD (n=86) | *p*-value |
| --- | --- | --- | --- |
| Ai/BSA (mm^2^/mm^2^) |  |  |  |
| 3rd-generation bronchi | 19.22 (7.32) | 22.96 (6.54) | 0.019 |
| 4th-generation bronchi | 5.41 (2.02) | 6.27 (1.98) | 0.052 |
| 5th-generation bronchi | 2.87 (1.19) | 3.68 (1.21) | 0.003 |
| 6th-generation bronchi | 1.51 (0.77) | 2.11 (0.84) | <0.001 |
| WT/√BSA (mm/mm) |  |  |  |
| 3rd-generation bronchi | 1.30 (0.11) | 1.22 (0.13) | <0.001 |
| 4th-generation bronchi | 1.13 (0.09) | 1.07 (0.10) | 0.002 |
| 5th-generation bronchi | 0.99 (0.08) | 0.95 (0.09) | 0.053 |
| 6th-generation bronchi | 0.86 (0.08) | 0.86 (0.09) | 0.646 |
| %WT (%) |  |  |  |
| 3rd-generation bronchi | 36.4 (5.1) | 33.4 (3.6) | 0.005 |
| 4th-generation bronchi | 40.3 (4.9) | 37.3 (4.5) | 0.006 |
| 5th-generation bronchi | 44.1 (4.7) | 41.0 (5.6) | 0.005 |
| 6th-generation bronchi | 47.5 (5.2) | 44.5 (4.9) | 0.011 |
| %LAA | 31.7 (7.9) | 38.3 (9.7) | <0.001 |

Data are expressed as mean (standard deviation).

^#^ACO were defined using criteria as follows: all three major criteria of 1) persistent airflow limitation (post-bronchodilator FEV_1_/FVC <0.70 or lower limit of normal) in individuals 40 years of age or older; 2) at least 10 pack-years of tobacco smoking or equivalent indoor or outdoor air pollution exposure; and 3) documented history of asthma before 40 years of age or bronchodilator response of >400 mL in FEV_1_: and at least one minor criterion of 1) documented history of atopy or allergic rhinitis; 2) bronchodilator response of FEV_1_ ≧200 mL and 12% from baseline values on 2 or more visits; or 3) peripheral blood eosinophil count of ≧300 cells/uL [39].

Ai, airway inner luminal area; WT, airway wall thickness; %WT, percentage of airway wall thickness; %LAA, percentage of low attenuation area < −950 HU; BSA, body surface area.

**Supplementary Table S11. Comparison of respiratory impedance between ACO using different criteria and COPD**

|  | ACO using different criteria^#^ (n=29) | Matched COPD (n=86) | *p*-value |
| --- | --- | --- | --- |
| Expiratory phase | | | |
| R5 (cmH_2_O/L/s) | 4.43 (2.61) | 3.47 (1.38) | 0.069 |
| R20 (cmH_2_O/L/s) | 3.26 (1.63) | 2.68 (0.90) | 0.081 |
| R5-R20 (cmH_2_O/L/s) | 1.17 (0.70) | 0.79 (0.64) | 0.076 |
| X5 (cmH_2_O/L/s) | −2.03 (2.84) | −1.06 (1.36) | 0.085 |
| Fres (Hz) | 13.27 (8.81) | 12.17 (6.64) | 0.542 |
| ALX (cmH_2_O/L/s×Hz) | 18.03 (28.62) | 8.11 (12.33) | 0.080 |
| Inspiratory phase | | | |
| R5 (cmH_2_O/L/s) | 3.86 (2.10) | 2.55 (0.96) | 0.003 |
| R20 (cmH_2_O/L/s) | 2.93 (1.26) | 2.20 (0.72) | 0.005 |
| R5-R20 (cmH_2_O/L/s) | 0.24 (0.40) | 0.35 (0.41) | 0.027 |
| X5 (cmH_2_O/L/s) | −1.46 (2.04) | −0.58 (0.63) | 0.030 |
| Fres (Hz) | 12.62 (6.80) | 9.65 (4.20) | 0.033 |
| ALX (cmH_2_O/L/s×Hz) | 11.03 (17.79) | 3.22 (4.80) | 0.0265 |
| Average of inspiratory and expiratory phase | | | |
| R5 (cmH_2_O/L/s) | 4.14 (2.33) | 3.01 (1.13) | 0.017 |
| R20 (cmH_2_O/L/s) | 3.09 (1.43) | 2.44 (0.78) | 0.025 |
| R5-R20 (cmH_2_O/L/s) | 1.05 (0.96) | 0.57 (0.50) | 0.015 |
| X5 (cmH_2_O/L/s) | −1.75 (2.35) | −0.82 (0.95) | 0.047 |
| Fres (Hz) | 12.94 (7.71) | 10.91 (5.24) | 0.194 |
| ALX (cmH_2_O/L/s×Hz) | 14.53 (15.89) | 5.66 (8.25) | 0.046 |
| Gap between inspiratory and expiratory phase | | | |
| R5 (cmH_2_O/L/s) | 0.57 (0.79) | 0.92 (0.71) | 0.036 |
| R20 (cmH_2_O/L/s) | 0.33 (0.53) | 0.49 (0.44) | 0.147 |
| R5-R20 (cmH_2_O/L/s) | 0.24 (0.40) | 0.44 (0.41) | 0.027 |
| X5 (cmH_2_O/L/s) | −0.57 (1.57) | −0.47 (0.94) | 0.754 |
| Fres (Hz) | 0.64 (3.14) | 2.52 (3.67) | 0.010 |
| ALX (cmH_2_O/L/s×Hz) | 7.00 (16.18) | 4.89 (8.82) | 0.507 |

Data are expressed as mean (standard deviation). ^#^ACO were defined using criteria as described in **Supplementary Table S9** [39]. R5, respiratory resistance at 5 Hz; R 20, respiratory resistance at 20 Hz: X5, respiratory reactance at 5 Hz; Fres, resonant frequency; ALX, low-frequency reactance area.

**Supplementary Table 12. Correlations between respiratory resistance and airway intraluminal area**

|  | R5_avg._ | | R20_avg._ | |
| --- | --- | --- | --- | --- |
|  | r | *p*-value | r | *p*-value |
| Ai/BSA |  |  |  |  |
| 3rd-generation bronchi | -0.415 | <0.001 | -0.415 | <0.001 |
| 4th-generation bronchi | -0.425 | <0.001 | -0.425 | <0.001 |
| 5th-generation bronchi | -0.410 | <0.001 | -0.395 | <0.001 |
| 6th-generation bronchi | -0.395 | <0.001 | -0.373 | <0.001 |

Data are expressed as Pearson correlation coefficient and *p*-value. Ai, airway inner luminal area; BSA, body surface area. R5, respiratory resistance at 5 Hz; R 20, respiratory resistance at 20 Hz: avg, an average of two breathing phase.
